# Gas Sensitivity and Sensing Mechanism Studies on Au-Doped TiO_2_ Nanotube Arrays for Detecting SF_6_ Decomposed Components

**DOI:** 10.3390/s141019517

**Published:** 2014-10-17

**Authors:** Xiaoxing Zhang, Lei Yu, Jing Tie, Xingchen Dong

**Affiliations:** State Key Laboratory of Power Transmission Equipment & System Security and New Technology, Chongqing University, Chongqing 400044, China; E-Mails: elaine@cqu.edu.cn (L.Y.); cherrytie0123@hotmail.com (J.T.); D.xc_cqu@outlook.com (X.D.)

**Keywords:** Au-doped TiO_2_ nanotube array, SF_6_ decomposed components, gas sensing response

## Abstract

The analysis to SF_6_ decomposed component gases is an efficient diagnostic approach to detect the partial discharge in gas-insulated switchgear (GIS) for the purpose of accessing the operating state of power equipment. This paper applied the Au-doped TiO_2_ nanotube array sensor (Au-TiO_2_ NTAs) to detect SF_6_ decomposed components. The electrochemical constant potential method was adopted in the Au-TiO_2_ NTAs' fabrication, and a series of experiments were conducted to test the characteristic SF_6_ decomposed gases for a thorough investigation of sensing performances. The sensing characteristic curves of intrinsic and Au-doped TiO_2_ NTAs were compared to study the mechanism of the gas sensing response. The results indicated that the doped Au could change the TiO_2_ nanotube arrays' performances of gas sensing selectivity in SF_6_ decomposed components, as well as reducing the working temperature of TiO_2_ NTAs.

## 1. Introduction

Sulfur hexafluoride (SF_6_) gas has excellent insulating and arc-extinguishing abilities and greatly improves the dielectric strength as an insulating medium. As a result, SF_6_ gas has been widely used in gas-insulated switchgear (GIS) [1–3]. A large number of domestic and international studies have indicated that partial discharge (PD) usually occurs and causes the decomposition of SF_6_ with trace moisture and oxygen into various products, such as SOF_4_, SOF_2_, SO_2_F_2_, SO_2_, H_2_S and HF [4,5], at the early stages of the electrical equipment insulation degradation. Such active gases produced by discharge energy will accelerate the aging of insulation and corrode metal surfaces, which may eventually lead to GIS failure. There have been no feasible and effective on-line monitoring methods so far.

Over the past decade, metal oxide semiconductor systems have obtained considerable achievements in sensing application, because of their specific surface geometry, unique functionality and various modification methods. For a recent review in the sensing field, metal doping, metal oxide synthesis and an innovative electrochemical structure are all utilized for the purpose of TiO_2_ modification [6]. Most of the literature in this area adopts resistive electrical sensors, where the active materials are powders or thin or films [7–10]. For example, Enrico *et al.* reported the gas sensing properties of TiO_2_-NiO thin films containing Au nanoparticles toward H_2_, CO, propane and H_2_S [11]. The metal modified TiO_2_ system is proven to have the potential to be an excellent chemical sensor for O_2_, H_2_, NO_2_ and NO [12–14]. Some theory studies based on first-principles calculation are also introduced to explore the surface interaction mechanism [15–17]. However, to the best of our knowledge, detections of SO_2_F_2_, as well as of SOF_2_ are rarely involved, and few examples of SO_2_ sensors upon metal-doped TiO_2_ have been reported.

Since the probable sensing mechanism involves the film pores and the surface oxygen adsorbed on component particles during the diffusion process, the morphology plays a crucial role in the sensing property. In order to explore the effect of the morphology on the sensing properties, several researchers have investigated this issue preliminarily. Existing achievements confirm that the gas sensitivity increases with decreasing crystal size as the quantum size effect and predict that crystals in different shapes would affect the sensitivity [18–20]. For example, Min-Hyun Seo *et al.* prepared TiO_2_ with different morphologies, nanoparticles and nanotubes on the basis of a hydrothermal treatment method with different treating temperatures [21]. However, other groups have mentioned that the accurate relationship between the electric resistance change of the sensor films and their morphology is a complex problem; the resistance change is not correlated well with their morphology [20,21]. This issue is a consequence of the fact that the electric resistance relies on various parameters, such as tube length, film thickness, grain size, crystal structure and physical factors, such as carrier density and effective mobility. According to our accomplished research on intrinsic [22] and Pt-doped [23] TiO_2_ nanotube array sensors (TiO_2_ NTAs) detecting SF_6_ decomposed components and previous achievements on common gases acquired by other research groups, a precious metal catalyst Au was chosen for this research. However, in the pure gold sensing application, the surface of pure Au is known to be unreactive to most gases, including CO and O_2_ [24]. While the quantum size effect in gold on a metal oxide support turns out to have an influence on the sensing performance, it is speculated that the reactivity of Au towards oxygen may depend on the small grain size of Au [25,26].

Herein, the present study is part of a systematic investigation of detections for SF_6_ decomposed products on different materials [27] for the purpose of setting up a practical sensing network for multi-gases detection. In this paper, nano-Au-deposited TiO_2_ NTAs were developed with the deposition-precipitation method. Then, gas sensing experiments were carried out to detect the main components of SF_6_ decomposition.

## 2. Experimental Section

### 2.1. Preparation of Au-TiO_2_ Nanotube Arrays

To prepare the Au-TiO_2_ nanotube arrays, the intrinsic TiO_2_ nanotubes were first fabricated and then Au was deposited onto the TiO_2_ nanotubes using the deposition-precipitation method. The intrinsic TiO_2_ nanotube arrays used in this paper were prepared by the anodic oxidation method [22], and NaOH was selected as the precipitating agent. Firstly, the pH of the 1.01 × 10^−3^ mol/L HAuCl_4_ solution was adjusted to 9 using NaOH solution. Then, the intrinsic TiO_2_ nanotubes were subsequently added to the above solution, resulting in the pH value decreasing. At the moment, a little more NaOH solution was needed to maintain the pH value at 9. Next, the resulting suspension was stirred for 2 h at 70 °C to allow the Au to be supported on the carrier sufficiently. During the process, the suspension became pale purple gradually, and the pH value remained at 9 after cooling. TiO_2_ nanotubes were picked out, washed, filtered, dried at room temperature and calcined for 4 h at 100 °C to finally obtain the Au-TiO_2_ nanotube sensors [28].

As for the reasons for keeping the pH at 9, the selection of a pH value of 8–9 is in agreement with several previous experimental investigations [29,30]. A number of groups have proven that the selection of pH leads to different geometries of TiO_2_ [31], and the pH of an aqueous solution dramatically affects the particle size of Au [32]. Though a finite value for the size cannot be deduced from the activity, since the electronic factors depend on the interaction with the support, the morphology of the particle or the chemical state of the gold, there is general agreement that the activity increases as the particle size decreases [33]. Hence, the pH value of the solution has significant influence on the catalytic activity. A low pH causes a big Au particle, while a high pH causes a low Au deposition amount. The optimum pH aims to not only cause Au to be completely precipitated, but also leads to an appropriate diameter [34]. Ivanova *et al.* have proven that when the pH value is above 8, the main species of Au in the solution is transformed from AuCl_4_^−^ to Au(OH)_4_, leading to a smaller particle diameter. In order to remove Cl^−^ ions completely [35], we chose a pH of 9.

### 2.2. Equipment and Methods for Gas Sensing Experiments of TiO_2_ NTAs

**Figure 1.**
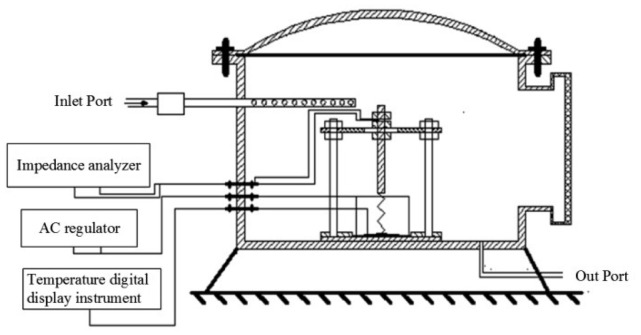
Detection test device in the TiO_2_ nanotube array (NTA) response measurements to SF_6_ decomposed components.

Figure 1 exhibits the detection test device in the TiO_2_ NTAs' response measurements to SF_6_ decomposed components. Standard gases of SF_6_ decomposed components were injected through the inlet port. A gas flow meter was used to control and detected the flow rate of the measured gas. A ceramic heater chip and thermal resistance probe were used to control and measure the surface temperature of the sensor. An impedance analyzer was utilized for recording the resistance value of the whole process. The relative changes of the sensor's resistance (*i.e.*, sensitivity) were calculated according to the following formula:

(1) $$R\%\;=\;\overset{\left(R-R_{0}\right)}{\;}/\underset{R_{0}}{\;}\times 100\%$$

where *R* represents the resistance value of the sensor after the detected gas injection and *R*_0_ indicates the resistance value in a N_2_ atmosphere. The response time of the sensor is defined as the time that the resistance change reaches 90% of the maximum.

Considering that TiO_2_ nanotube arrays have the ability to adsorb oxygen and water vapor in the air, a dynamic method was introduced in this experiment to exclude the impact of this factor [36]. The specific steps were as follows.

Firstly, the TiO_2_ nanotube sensor was placed on the ceramic heating chip, and then, the relative position of the two electrodes and sensors was adjusted, making the two platinum chips full touch the sensor with moderate intensity. Confined space was required in cylinder, and the vacuum pump was turned on to ensure that no sensitive gases remained in the cylinder. High purity N_2_ at a flow rate of 0.1 L/min along with the heating power on the sensor were appropriate. Besides, the sensor surface temperature was set to a pre-designed operating temperature by adjusting the regulator. Then, the stable resistance of the TiO_2_ NTAs was recorded as *R*_0_.

Secondly, one of the decomposed components of SF_6_ was pumped in, such as SO_2_, at the same flow rate as N_2_. At that moment, the sensor resistance changed dramatically and quickly stabilized (fluctuating around a resistance), which was recorded as the final response resistance.

Finally, after the resistance of the sensor was stable, the high-purity N_2_ flowed in at a flow rate of 0.1 L/min until the sensor resistance was gradually stabilized at a certain value again, which was recorded as *R*'_0_.

## 3. Results and Discussion

### 3.1. Morphological Characterization and Analysis of Au-TiO_2_ Nanotube Arrays

The sample morphology was analyzed by scanning electron microscopy (SEM). The SEM images were obtained by JEOLJSM7000 field emission SEM equipment operated at 10 kV.

**Figure 2.**
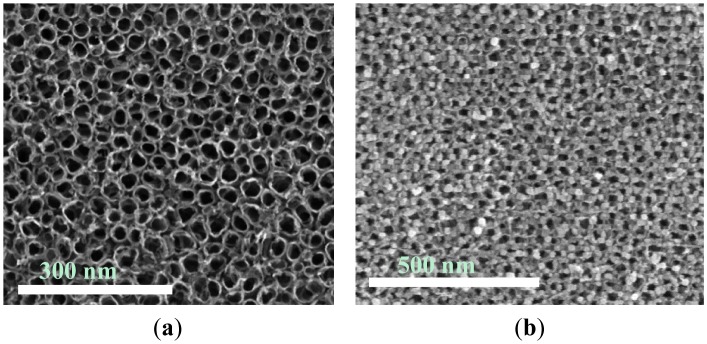
(**a**) SEM image of the intrinsic TiO_2_ nanotubes. (**b**) SEM image of the Au-TiO_2_ nanotubes.

Figure 2 shows SEM images of the pore size distribution of films composed of (a) intrinsic TiO_2_ nanotubes and aggregates and (b) the Au nanoparticles distribution of Au-TiO_2_ prepared by the deposition-precipitation method. The surfaces of the films were observed before and after Au deposition. It is obvious that the morphology of the TiO_2_ films is significantly changed after the Au nanoparticle modification. The adopted fabrication method results in the formation of tubular TiO_2_ of 25 nm in diameter. After the Au deposition treatment, the diameter of the tubes remains about the same. However, on the Au-TiO_2_ surface, the pipes are covered with Au nanoparticles of a dozen nanometers in size, aggregating at the pipe orifices. The SEM images confirm that the formed films, whether composed of intrinsic or Au-TiO_2_, are homogeneous with a uniform distribution of pores or Au nanoparticles, respectively, as expected.

**Figure 3.**
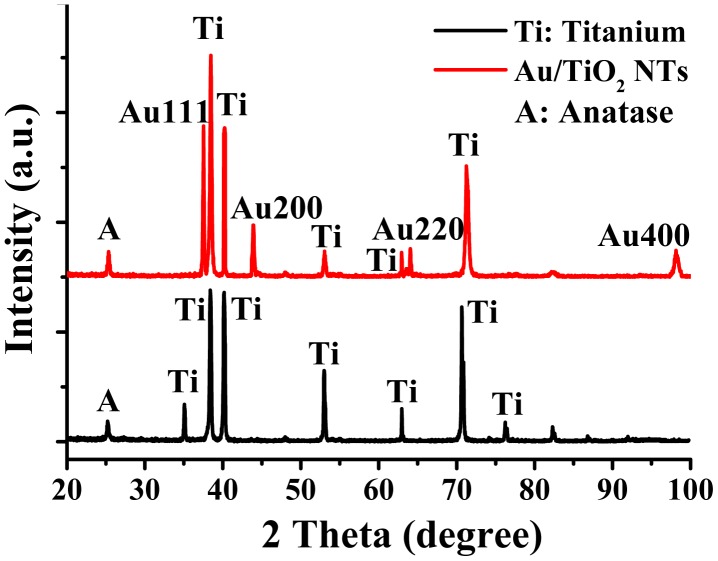
XRD of Au-TiO_2_ nanotubes.

The crystal structures of the obtained intrinsic TiO_2_ and Au-TiO_2_ nanotubes were analyzed by X-ray diffraction, measured on an X'pert Pro (PANalytical, The Netherland) using Cu Kα radiation (λ = 0.15405 nm) at 40 kV, 35 mA. The wide-angle XRD patterns were collected at a scanning speed of 10°/min over the 2θ range of 20°–100°. Figure 3 gives the XRD patterns of the products prepared by the deposition-precipitation treatment. Previously, Varghese *et al.* observed both the anatase and rutile phases of TiO_2_ by annealing treatment in ambient oxygen [37,38]. In our study, the labels A at 25.3° are observed in intrinsic, as well as in Au-doped TiO_2_, indicating that the crystal phases of TiO_2_ are both anatase according to previous structural characterizations [39], for which it can be confirmed that, in these preparation conditions, the TiO_2_ nanotubes adopt an anatase crystal structure, while a rutile structure is not observed. The labels T and Au××× represent the reflections from the titanium substrate and different Au crystallographic forms. It is clearly seen from Figure 3 that characteristic gold peaks come into existence in XRD analysis observed at 38.2° (111), 44.2° (200), 64.3° (220) and 98.1° (400), respectively. The main Au (111) characteristic peak suggests that approximately 10-nm gold nanoparticles are coated onto the anodized TiO_2_ nanotubes on the basis of the Scherer formula [40]. Meanwhile, a certain amount of 200, 220 and 400 Au particle crystal forms do exist.

### 3.2. Effects of Different Working Temperatures on the Gas Sensing Properties of Au-TiO_2_ NTAs

There is a popular belief that the characteristics of a metal oxide semiconductor are greatly affected by the doped metal or metalloid, which would also influence the operating temperature in a sensing application further. Therefore, it is necessary to investigate the gas sensing response of Au-TiO_2_ NTAs to SF_6_ decomposed components (*i.e.*, 50 ppm SOF_2_, SO_2_F_2_ and SO_2_) in an operating range of 20 °C to 200 °C in order to find out the optimum operating temperature.

**Figure 4.**
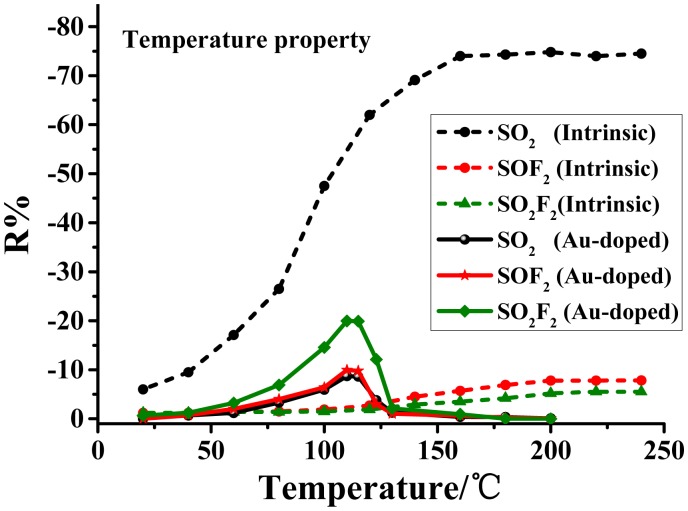
Sensor responses for 50 ppm SO_2_ (black dots), SOF_2_ (red dots) and SO_2_F_2_ (green dots), respectively, for the intrinsic (short dashed lines, in the range of 20 °C∼240 °C) and Au-doped TiO_2_ (solid lines, in the range of 20 °C∼200 °C) nanotubular films at different working temperatures.

Figure 4 depicts the curves of the resistance changes' rate (*i.e.*, the response value) of Au-doped and intrinsic TiO_2_ NTAs to SO_2_, SOF_2_ and SO_2_F_2_ at different operating temperatures. The response value of the intrinsic TiO_2_ nanotube to SF_6_ decomposed components increases as the surface temperature rises, reaching saturation around 180 °C, which is considered the optimum operating temperature. In the case of Au-TiO_2_, the resistance response increases with increasing operating temperature before 110 °C, following the typical behavior of an oxide semiconductor. However, the resistance response dramatically drops down when the temperature exceeds 110 °C. Hence, the optimum operating temperature of the Au-TiO_2_ nanotube sensor is taken as 110 °C. A comparison of Au-doped and intrinsic TiO_2_ indicates that Au-doping reduces the working temperature of TiO_2_ NTAs along with obvious changes in the temperature characteristic curve.

The performance of the intrinsic TiO_2_ nanotube sensors maintaining its response value after it reaches 180 °C might be attributed to the dynamic equilibrium of the gas adsorption and desorption rate on the sensor's surface in the meantime. As for Au-TiO_2_, the Au nanoparticles change the microscopic structure and charge distribution of the surface, and the doped Au results in a promoted chemical desorption rate when the temperature surpasses 110 °C , causing the oxygen desorption rate to be faster than its adsorption rate. As a result, the oxygen chemisorption density on the surface decreases, leading to a rapid drop of the response value [23].

### 3.3. Sensing Performances of Au-TiO_2_ NTAs for Detecting SF_6_ Decomposed Components

The gas sensing response curves of SO_2_, SOF_2_ and SO_2_F_2_ for Au-TiO_2_ NTAs were recorded at different concentrations (*i.e.*, 25 ppm, 50 ppm, 75 ppm, 100 ppm) under the optimal operating temperature (110 °C). The results were linearly fit to investigate the linear relationship between the sensor's resistance change and the gas concentration. Therefore, the concentration of target gases in real power equipment could be estimated through the linear relationship acquired by these sample gases.

#### 3.3.1. Sensing Performances of Au-TiO_2_ NTAs for SO_2_

**Figure 5.**
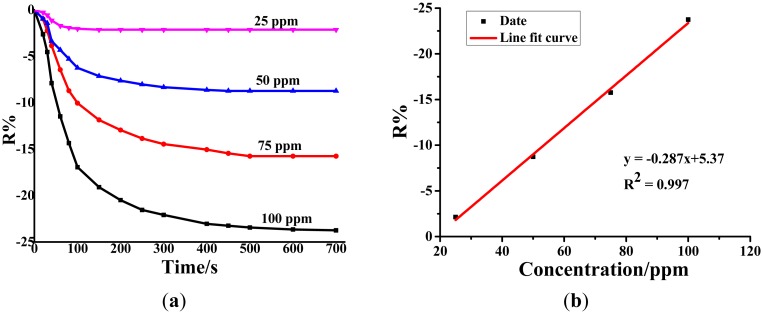
(**a**) Au-TiO_2_ NTAs' response to different concentrations of SO_2_ at the 110 °C working temperature. (**b**) Linear relationship between the sensor's response value and the SO_2_ concentration.

As Figure 5 shows, the resistance change rates of the Au-TiO_2_ nanotube gas sensor for SO_2_ at 25 ppm, 50 ppm, 75 ppm and 100 ppm are −2.14%, −8.73%, −15.76% and −23.75%, respectively. The linear relationship between the sensor's resistance change rate and the SO_2_ concentration is fitted as y = −0.287x + 5.37 with a linear correlation coefficient (*R*^2^) of 0.997.

#### 3.3.2. Sensing Performances of Au-TiO_2_ NTAs for SOF_2_

**Figure 6.**
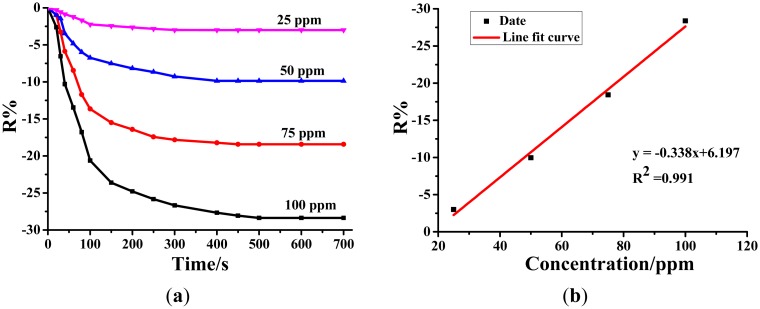
(**a**) Au-doped TiO_2_ NTAs' response to different concentrations of SOF_2_ at the 110 °C working temperature. (**b**) Linear relationship between the sensor's response value and the SOF_2_ concentration.

Figure 6 exhibits the sensing response curves of the Au-TiO_2_ nanotube sensor for SOF_2_ at different concentrations under 110 °C. From Figure 6a, the resistance change rates that correspond to 25 ppm, 50 ppm, 75 ppm and 100 ppm of SOF_2_ are separately −3.00%, −9.97%, −18.42% and −28.37%. After linear fitting, the linear function is calculated to be y = −0.338x + 6.197, as shown in Figure 6b, with *R*^2^ equaling 0.991. It can be concluded that, within a certain range of concentrations, a linear relationship between the resistance change rate of the Au-TiO_2_ nanotube sensor and the SOF_2_ concentration is also displayed.

#### 3.3.3. Sensing Performances of Au-TiO_2_ NTAs for SO_2_F_2_

**Figure 7.**
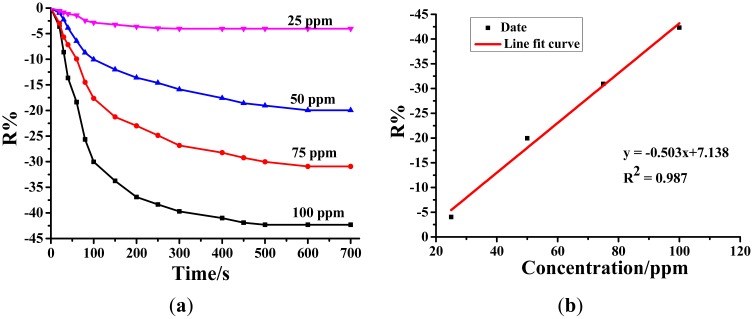
(**a**) Au-doped TiO_2_ NTAs response to different concentrations of SO_2_F_2_ at the 110 °C working temperature. (**b**) Linear relationship between the sensor's response value and the SO_2_F_2_ concentration.

Resistance change rates of Au-TiO_2_ nanotube gas sensor for SOF_2_ with different concentrations at 25 ppm, 50 ppm, 75 ppm and 100 ppm are respectively −4.04%, −19.58%, −30.93% and −42.31%, as shown in Figure 7a. The linear fitting relationship is y = −0.503x + 7.13,8 and the linear correlation coefficient *R*^2^ equals 0.991.

### 3.4. Selective Gas Sensing Performances of Au-TiO_2_ NTAs

**Figure 8.**
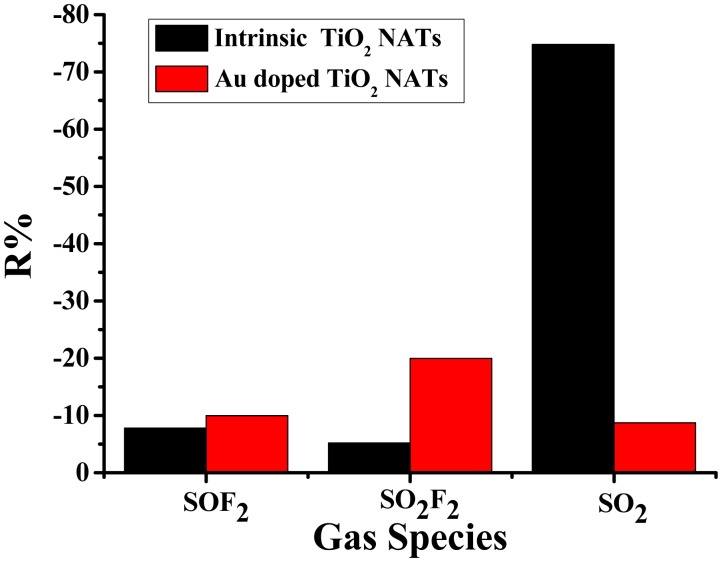
Sensor responses of Au-doped and intrinsic TiO_2_ nanotube arrays for SF_6_ decomposition components.

Figure 8 shows the gas sensing response comparison chart of intrinsic and Au-doped TiO_2_ NTAs at their optimum operating temperatures for 50 ppm SF_6_ decomposed gases, *i.e.*, SO_2_, SOF_2_ and SO_2_F_2_, where the gas sensing properties of intrinsic TiO_2_ NTAs have been discussed in [22]. The responses of intrinsic and Au-doped TiO_2_ NTAs both exhibit a negative behavior, *i.e.*, the resistances of intrinsic and Au-doped TiO_2_ NTAs decrease after introducing these gases.

The gas sensing response values of the intrinsic TiO_2_ nanotube sensor are SO_2_ (−74.6%) > SOF_2_ (−7.82%) > SO_2_F_2_ (−5.52%), while for the Au-TiO_2_ nanotube sensor are SO_2_F_2_ (−19.95%) > SOF_2_ (−9.97%) > SO_2_ (−8.73%). It is worth noting that the experimental results in our study are statistically significant, the values of which are at the average level according to dozens of experiments. Obviously, the response value of SO_2_F_2_ dramatically increases, while SO_2_ is reduced, and the response of SOF_2_ remains constant. The selective detection of SO_2_F_2_ was actually achieved in our experimental research by the modification of Au nanoparticles at the appropriate operation temperature. Hence, the Au-TiO_2_ NTAs are potential substrates for the SO_2_F_2_ detection application. Furthermore, combined Au-doped and intrinsic TiO_2_ arrays are promising substrates for SF_6_ decomposition component detection.

### 3.5. Stability Investigation of Au-TiO_2_ NTAs

**Figure 9.**
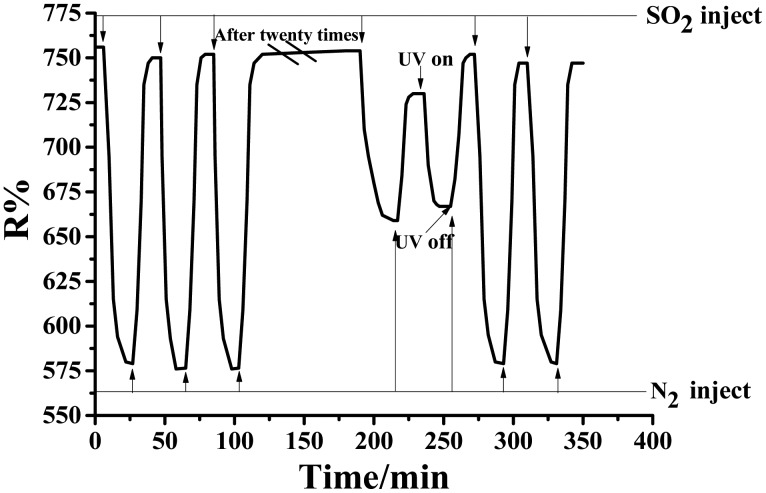
Recovery curve of the Au-TiO_2_ nanotubes sensor.

Serious repeated experiments of SO_2_ detection at 110 °C were carried out to study the anti-sulfuration ability of a noble metal catalyst for stability investigation. The experimental results are depicted in Figure 9. At the beginning, after 100 ppm SO_2_ gas is introduced, apparent changes in the sensor's resistance occur. The pure N_2_ is injected after the sensor's resistance becomes stable, and then, the resistance gradually restores to the initial value. Once the resistance of the sensor exposure to N_2_ becomes stable, two more times detection procedures are needed continuously. The sensitivity is found to be unchanged along with the resistance value, which always returns to the initial one. This observation indicates that the sensing behavior of Au-TiO_2_ NTAs prefers a reversible interaction with SO_2_. When the experiment repeatedly detects SO_2_ about 20 times, the reduced sensitivity of the sensor is observed. Not even pure N_2_ can restore the resistance to the original one yet. Then, UV irradiation is adopted; the sensor's resistance rapidly decreases again and remains at a value below the initial resistance when it reaches stability. N_2_ is introduced again and makes the resistance value gradually increase, to achieve stability ultimately. When SO_2_ passes into the equipment, the gas sensor's sensitivity obtains the same level as the initial detection. When the Au-TiO_2_ nanotube sensor is utilized to test the other two gases, its stability curve is basically the same as that of SO_2_, which will not be displayed here again. The present work demonstrates that the Au-TiO_2_ in this method provides reusable features, due to its self-cleaning property [41].

As can be concluded, sulfur has certain toxicity for the Au-TiO_2_ nanotube sensor, which will seriously affect the initial resistance and the sensitivity of the sensor. This obstacle has proven to be effectively solvable by UV irradiation to make the S ions desorb. Compared with the results in the literature [23], the repeatability of Au-doped sensors is greater than that of Pt-doped TiO_2_ sensors. The gas sensing property begins to decline when Au-TiO_2_ is repeatedly exposed to SO_2_ about 20 times, while in the Pt-TiO_2_ case, it immediately presents an inactive state after the second time, which demonstrates a significant sulfur poisoning phenomenon. Hence, from the experimental observations, we speculate that the anti-sulfide ability of Au is stronger than that of Pt. In all, a comparison of the recovery curves of Au-doped and Pt-doped TiO_2_ sensors reveals that Au-doped sensors have better repeatability and a superior ability of anti-sulfurization, which is due to the fact that it is more difficult for Au to bond to S compared to Pt [42]. Therefore, Au-doped sensors exhibit better stability, which is a promising property in sensing applications.

### 3.6. Gas Sensing Mechanism Study of Au-TiO_2_ NTAs

In order to find out the sensing mechanism, we will discuss the nature of the bonding between Au on TiO_2_ in the first place. Theoretical studies have been done to find out the deep principle of Au and TiO_2_. Rodriguez *et al.* have found essentially covalent bonding between Au and Ti sites [43]. Besides, a minor depletion of electrons on Au was observed when Au was bonded to O centers on the surface [44,45]. Moreover, previous research indicates that gold grows on TiO_2_ epitaxially, generating two- or three-dimensional particles (Volmer–Weber growth mode) [24,46], which is in accordance with our SEM analysis. A general agreement has been reached that two key phenomena are basically responsible for the prominent sensitivity found for Au-TiO_2_ in our experimental study and previous catalytic investigations. On the one hand, interactions with TiO_2_ electronically perturb gold, making it more chemically active [47–49]. On the other hand, gold increases the concentration of O vacancies at the surface of the oxide [50], enhancing the chemical activity of TiO_2_, as well.

As shown in Figure 8, the gas sensing response values of intrinsic and Au-doped TiO_2_ nanotube sensors for SO_2_, SOF_2_ and SO_2_F_2_ are concurrently negative responses. According to the existing achievements, three measured gases all play the role of an electron donating gas. The following reaction occurs:

(2) $$R+{O_{\text{ads}}}^{-}\Leftrightarrow RO_{\text{ads}}+e^{-}$$

R represents the resistance of SO_2_, SOF_2_ and SO_2_F_2_, three SF_6_ decomposed components; *O_ads_*^−^ denotes the adsorbed oxygen on the sensor's surface.

The charge transfer that results in the negative responses is important during the processes occurring at the gases/oxide interfaces. Oxygen ions at the TiO_2_ boundaries between the grains cause a higher potential barrier, which will block the charge carriers' transfer, leading to a relatively large resistance [51]. When a reducing gas or an electron donating gas comes into contact with the TiO_2_ nanotube sensor, the chemical reaction of the gases and the adsorbed oxygen on the sensor's surface occurs, resulting in a sharp decrease of the adsorbed oxygen. Thereby, the potential barrier of the grain boundary on the surface is reduced and then contributes to more charge carrier transfers and a decrease in the resistance of the TiO_2_ nanotube sensor to obtain the sensing response.

A detailed understanding of the processes occurring at the Au/oxide interfaces and their relationship to the sensing performance of sensor devices could lead to enhancements in the selective phenomenon displayed in Figure 8, where the SO_2_F_2_ response value increases greatly, while that of SOF_2_ remains essentially unchanged and the response for SO_2_ decreases. This is probably a consequence of poisoning effect of sulfur (being less sensitive to the presence of S-containing molecules) for a noble metal on the sensing performances. A fundamental understanding of the chemistry of S-containing molecules, like CH_3_SH, H_2_S and S_2_, on a metal/oxide surface (Al_2_O_3_, ZnO, Cu_2_O *etc.*) has been achieved [52,53]. On the surface of a metal oxide, sulfur prefers to interact with the supported metal sites rather than the oxide support, producing sulfide or sulfate that has a different electronic property [54]. The toxicity order of these three gases is SO_2_ > SOF_2_ > SO_2_F_2_, according to [55]. Sulfate produced by the adsorption of SO_2_ blocks the electron holes on TiO_2_, causing a reduction of O_2_ or O^2−^ adsorption on the vacancies. The strongest poisoning effect of SO_2_ might account for the detrimental situation for SO_2_ detection.

The surface adsorption-controlled mechanism [56] for the Au/TiO_2_ surface interactions to these three SF_6_ components is another reason for this selectivity case. For TiO_2_ semiconductor-based gas sensors, target gases diffuse on the TiO_2_ sensing film through pores and interact with surface oxygen, as well as adsorbed Au particles, to induce the electronic resistance change [57]. The concentration of these gases decreases inside the TiO_2_ film as a consequence of the diffusion. The doped Au particles provide more chemically active sites on the TiO_2_ surface, while making the TiO_2_ film be insufficiently porous at the same time. We have found that the intrinsic TiO_2_ NATs have a remarkable sensitivity to SO_2_ in our previous published research [22]. The porosity of intrinsic TiO_2_ is found to be the dominant reason for the pronounced sensitivity of SO_2_. Such macropores of intrinsic TiO_2_ provide effect-diffusivity paths for molecules and enhance the utility factor of the sensing film. Besides, the insensitivity of intrinsic TiO_2_ to SO_2_F_2_ and SOF_2_ is probably because they are difficult to diffuse deep inside the TiO_2_ films with relatively large molecular sizes [21,58]. However, in this work, the Au nanoparticles cover the efficient porous morphology, which leads to a decrease in the sensor response for SO_2_, due to a decrease in the utility factor of the TiO_2_ sensing film or a decrease in the accessibility of this gas. Hence, the doping is actually disadvantageous for SO_2_ detection. However, things change to another way when it comes to SO_2_F_2_ upon Au-TiO_2_. At the optimum temperature, Au exhibits an active catalytic activity that leads to a rupture of the S-F bonds. A similar phenomenon of SO_2_F_2_ dissociation has been observed at the crystal oxygen vacancy on the TiO_2_ surface through the first-principles calculations [59]. Thus, the chemisorption effect of Au-TiO_2_ for SO_2_F_2_ is a reliable reason that accounts for the experimental response. As for the SOF_2_ adsorption case, from the experimental observation, the interaction between Au-TiO_2_ and SOF_2_ is not as strong as the SO_2_F_2_ case. In all, we have reasons to speculate that the poisoning effect and the surface adsorption-controlled mechanism induce the selective properties in sensing performances.

## 4. Conclusions

The detection of SF_6_ decomposed gases is becoming ever more important due to the significant relationship with insulation faults in power equipment. Current methods of detection suffer from the shortage of on-line monitoring. Thus, there is value in developing an effective method for detection. The electrochemistry sensor is proven to be a promising path to achieve on-line detection.

Here, SF_6_ decomposed gas sensors incorporating Au and TiO_2_ were fabricated through the deposition-precipitation method and studied under a spectrum of different operating temperatures. The study of the sensing response of Au-TiO_2_ sensors reveals the dependency on the optimum temperature. These Au-TiO_2_ sensors were capable of detecting 50 ppm SO_2_F_2_, SOF_2_ and SO_2_ with reproducible performances. Meanwhile, the responses for SO_2_F_2_, SOF_2_ and SO_2_ at the optimum temperature (110 °C) indicate selectivity for SO_2_F_2_ detection, which we attribute to the poisoning effect of SO_2_ and the surface adsorption-controlled mechanism of all gases at the Au/TiO_2_ surface. A comparative study of the optimum operation temperature of intrinsic TiO_2_ and Au-TiO_2_ reveals that the doped Au nanoparticles reduce the working temperature. In addition, the differences in the recovery curves of Au-doped and Pt-doped TiO_2_ nanotube sensors confirm that Au-doped TiO_2_ has a better anti-sulfuration ability and stability.

In the future, we aim to evaluate the relationship between the morphology of the Au-TiO_2_ composite and the sensing properties, since the morphology was considered to have a significant effect on the sensor response in previous research. We believe that this could make the method of producing SF_6_ decomposed gas sensors viable in practical application.
